# Association Between Chikungunya Infection and Rheumatoid Arthritis in Taiwan—A Cross‐Sectional Study

**DOI:** 10.1111/1756-185x.70784

**Published:** 2026-08-05

**Authors:** Chang‐Yi Yen, Chang‐Chi Lin, Ping‐Chang Lin, Ching‐Yi Tsai, Li‐Teh Liu, Chen‐Hsuan Lin, Chun‐Hong Chen, Chia‐Chun Tseng, Hua‐Chen Chan, Cheng‐Chin Wu, Tsan‐Teng Ou, Jih‐Jin Tsai, Jeng‐Hsien Yen

**Affiliations:** ^1^ Division of Allergy, Immunology and Rheumatology, Department of Internal Medicine Kaohsiung Medical University Hospital Kaohsiung Taiwan; ^2^ Institute of Medical Informatics, College of Electrical Engineering and Computer Science National Cheng Kung University Tainan Taiwan; ^3^ Institute of Preventive Medicine National Defense Medical Center Taipei Taiwan; ^4^ Department and Graduate Institute of Microbiology and Immunology National Defense Medical Center Taipei Taiwan; ^5^ Tropical Medicine Center, Kaohsiung Medical University Hospital Kaohsiung Taiwan; ^6^ Department of Medical Laboratory Science and Biotechnology College of Medical Technology, Chung‐Hwa University of Medical Technology Tainan City Taiwan; ^7^ School of Medicine, College of Medicine, Kaohsiung Medical University Kaohsiung Taiwan; ^8^ National Mosquito‐Borne Diseases Control Research Center National Health Research Institutes Zhunan Township Miaoli County Taiwan; ^9^ National Institute of Infectious Diseases and Vaccinology, National Health Research Institutes Zhunan Township Miaoli County Taiwan; ^10^ Department of Medical Laboratory Science College of Medical Science and Technology, I‐Shou University Kaohsiung Taiwan; ^11^ Division of Infectious Diseases, Department of Internal Medicine Kaohsiung Medical University Hospital Kaohsiung Taiwan; ^12^ Graduate Institute of Clinical Medicine College of Medicine, Kaohsiung Medical University Kaohsiung Taiwan

**Keywords:** chikungunya arthritis, chikungunya virus, neutralizing antibody, rheumatoid arthritis

## Abstract

**Objectives:**

Chikungunya virus (CHIKV) infection might cause chronic chikungunya arthritis resembling rheumatoid arthritis (RA). The purpose of this study is to investigate the potential role of this virus in the pathogenesis of RA in Taiwan, a non‐endemic area.

**Methods:**

This cross‐sectional study enrolled 98 RA patients and 200 healthy controls. Serum CHIKV IgM and IgG were tested using two commercial enzyme‐linked immunosorbent assay (ELISA) kits (Abcam and Euroimmun) and an in‐house neutralization assay. ELISA‐IgM was performed only in patients with RA. ELISA or neutralization positive samples were further tested for CHIKV RNA by reverse‐transcription‐polymerase chain reaction. Associations between CHIKV seropositivity and various autoantibodies were analyzed. The agreements among these assays were also evaluated.

**Results:**

Neutralizing antibodies were detected in 2 of 98 RA patients and in none of the controls (*p* = 0.107, Fisher's exact test; *p* = 0.043, chi‐square). Both patients were absent from Abcam‐IgM and ‐IgG, as well as Euroimmun‐IgM and ‐IgG testing. There was no association between Abcam‐IgM or Euroimmun‐IgM and rheumatoid factor, anti‐cyclic citrullinated peptide, antinuclear, or anti‐extractable nuclear antigen antibodies. Poor agreements were noted between the neutralizing assay and Abcam‐IgG or Euroimmun‐IgG (*κ* = 0 and 0, respectively). A similar finding was also found between Abcam‐IgM and Euroimmun‐IgM (*κ* = −0.38).

**Conclusion:**

Our study was not sufficiently powered to establish a causal association between CHIKV infection and RA development. Poor agreements were noted among different commercial ELISA kits and neutralization assays, underscoring the limitations of the serology assay.

## Key Points

1. Our study was insufficiently powered to establish a definitive association between CHIKV infection and RA development due to low prevalence.
2. Testing results should be interpreted judiciously because of poor agreement among different assays.

## 1 Introduction

Rheumatoid arthritis (RA) is a chronic inflammatory systemic autoimmune disease characterized by synovial inflammation, progressive joint damage, and long‐term disability, with possible extra‐articular involvement. The etiology is multifactorial, involving genetic predisposition, sex, and environmental factors, including smoking and viral infections such as parvovirus B19 (as suggested by our previous studies [1, 2]), retrovirus, and Epstein–Barr virus [3].

Chikungunya is a mosquito‐borne disease caused by the chikungunya virus (CHIKV) infection. Acute CHIKV infection typically presents with fever, headache, rash, and debilitating arthralgia that resolves within weeks of infection in most cases, but a subset of patients develops chronic polyarthritis lasting months to years [4]. Imaging of the affected joints in these patients demonstrates synovial inflammation, pannus formation, and bone erosion resembling RA [5, 6, 7]. Epidemiological studies have shown that up to 10%–30% of CHIKV‐infected individuals meet the classification criteria for RA [8, 9]. Moreover, being female and smoking are risk factors for developing chronic chikungunya arthritis, as in RA [10, 11, 12].

Similar to RA, chronic chikungunya arthritis can be effectively treated using conventional disease‐modifying anti‐rheumatic drugs (DMARDs) such as methotrexate, hydroxychloroquine, sulfasalazine, or biologics such as etanercept or adalimumab [13, 14].

Immunological studies further reveal a significant overlap between CHIKV‐induced arthritis and RA, including shared proinflammatory cytokine patterns, matrix metalloproteinase, immune cell infiltration, and autoantibody profiles [15, 16, 17, 18, 19]. The transcriptomic analysis also shows a connection between CHIKV infection and RA [20].

Although convincing evidence links CHIKV infection to chronic arthritis and RA development, most of these studies were performed in endemic areas, and a similar study in non‐endemic areas is still lacking. There are several methods to detect the CHIKV antibody, and the accuracy of commercial kits versus the neutralization test, a standard method, remains uncertain, particularly in autoimmune patients prone to cross‐reactivity and false positivity [21].

The purpose of this study is to investigate whether CHIKV infection plays a role in the development of RA in Taiwan, a non‐endemic area, and to compare the consistency of these measurement methods.

## 2 Materials and Methods

### 2.1 Study Design and Participants

We conducted a cross‐sectional study at Kaohsiung Medical University Hospital (KMUH), Taiwan. A total of 98 patients with established RA and 200 healthy controls were enrolled from 2020 to 2022, with all RA patients meeting the 2010 EULAR‐ACR classification criteria [22]. Written informed consent was obtained from all participants, while the study was approved by the KMUH Institutional Review Board (KMUHIRB‐E(I)‐20 200 061) and conducted in accordance with the Declaration of Helsinki.

### 2.2 Rheumatologic Marker Testing

Rheumatoid factor (RF) was measured with the Siemens N Latex RF kit (Siemens Healthcare Diagnostics, Erlangen, Germany). Anti‐cyclic citrullinated peptide (anti‐CCP) and anti‐extractable nuclear antigen (anti‐ENA) antibodies were detected using the EliA CCP and EliA Symphony kits, respectively (Phadia AB, Uppsala, Sweden). Antinuclear antibodies (ANA) were assessed by the indirect immunofluorescence method (Euroimmun, HEp‐20‐10 EUROPattern).

### 2.3 CHIKV Antibody Testing and Neutralizing Antibody Assay

CHIKV IgM and IgG were tested using Abcam Human ELISA Kits (Abcam, Cambridge, UK) and Euroimmun ELISA Kits (Euroimmun, Lübeck, Germany). IgM testing was performed only in RA patients, not in healthy controls, because healthy controls were typically free of any symptoms or signs of chikungunya infection, and Taiwan is a non‐endemic area. In Euroimmun kits, the recombinant structural protein of CHIKV was used as the antigen with a ratio of the extinction value of the sample over the extinction value of the calibrator being calculated. The ratios ≧ 1.1, ≧ 0.8 to < 1.1, and < 0.8 were interpreted as positive, borderline, and negative, respectively. In Abcam kits, CHIK viral antigen was used to detect the anti‐chikungunya antibody with the standard units of > 11, 9–11, and < 9 being interpreted as positive, gray zone, and negative, respectively. Neutralizing antibodies were detected using an in‐house assay developed by Lin et al. [21].

### 2.4 CHIKV RNA Detection

Reverse transcription‐polymerase chain reaction (RT‐PCR) was performed on samples positive for CHIKV IgM or neutralizing antibodies to evaluate whether the subjects had an ongoing infection. The SYBR Green assay was performed to detect CHIKV genomic RNA. This assay targeted the E1 gene using specific primers recommended by the Taiwan Centers for Disease Control: CHIK‐forward (5′‐AAGCT YCGCGTCCTTTACCAAG‐3′) and CHIK‐reverse (5′‐CCAAATTGTCCYGGTCT TCCT‐3′). A clinical sample was defined as CHIKV‐positive if the cycle threshold (Ct) value was ≤ 35 and the melting temperature (Tm) was ≥ 79°C. Samples with a Ct > 35 or Tm < 79°C were considered negative.

### 2.5 Statistical Analysis

For statistical testing, the SPSS software (IBM Corp., Armonk, NY) was used. The *t*‐test was used to compare the age difference between RA patients and controls, while the chi‐square or Fisher's exact test was used to evaluate the associations between categorical variables. Odds ratios (OR) and 95% confidence intervals (CI) were estimated using the Haldane–Anscombe correction to account for zero cell counts, with Cohen's *κ* being used to assess the agreement between different assays. Statistical significance was defined as *p* < 0.05.

## 3 Results

### 3.1 Demographic Characteristics of RA Patients and Healthy Controls

The *t*‐test and chi‐square tests were used to evaluate the differences in age and sex distributions, respectively, in RA patients and controls (Table 1). There were no significant differences between these two groups.

**TABLE 1:** Demographic characteristics of RA patients and controls.

	Age (mean ± SD, years)	95% CI	*p*	Sex (Female: Male)	OR (95% CI)	*p*
RA (*n* = 98)	51.70 ± 13.96	−0.400~5.889	0.087	76: 22	0.974 (0.545~1.741)	0.930
Controls (*n* = 200)	48.96 ± 10.07			156: 44		

*Note:* The *t*‐test was used for statistical analysis of the age difference between RA patients and controls. The chi‐square test was used for statistical analysis of sex distribution between RA patients and controls.

Abbreviations: CI, confidence interval; OR, odds ratio; RA, rheumatoid arthritis.

### 3.2 Chikungunya Antibody and RT‐PCR Testing Results in RA Patients and Healthy Controls

There was no significant difference in Euroimmun‐IgG seropositivity between RA patients and controls (*p* = 0.476; Table 2). Two of 98 RA patients had positive neutralizing‐IgG antibody in comparison to none of 200 controls (Fisher's exact test, *p* = 0.107; Haldane–Anscombe correction, OR = 10.4, 95% CI = 0.5–217; chi‐square test, *p* = 0.043). Although the OR was high, statistical significance could not be obtained due to the low number of cases with positive neutralizing‐IgG antibodies. No IgM testing was performed in controls. RT‐PCR was further performed on all samples that were positive for Abcam‐IgM, Euroimmun‐IgM, or neutralization‐IgG, and all yielded negative results (data not shown).

**TABLE 2:** Chikungunya antibody testing results in RA patients and controls.

	RA (*n* = 98)	Control (*n* = 200)	OR (95% CI)	*p*
Abcam (ELISA)‐IgM				
Positive	7	NA		NA
Gray zone	14	NA		
Negative	77	NA		
Abcam (ELISA)‐IgG				
Positive	0	0		NA
Gray zone	0	0		
Negative	98	200		
Euroimmun (ELISA)‐IgM				
Positive	2	NA		NA
Borderline	0	NA		
Negative	96	NA		
Euroimmun (ELISA)‐IgG				
Positive	0	1		0.476
Borderline	0	2		
Negative	98	197		
Neutralizing‐IgG				
Positive	2	0	10.4 (0.5–217)	0.107^a^
Negative	96	200		

*Note:* IgM tests were not performed in controls.

Abbreviations: CI, confidence interval; NA, not applicable; OR, odds ratio; RA, rheumatoid arthritis.

^a^ Fisher's exact test was used for statistical analysis, and Haldane–Anscombe correction was used to measure the OR and 95% CI. The *p*‐value would be 0.043 if the chi‐square test was applied.

### 3.3 Association Between CHIKV IgM Positivity and Autoantibodies in RA

Abcam‐IgM and Euroimmun‐IgM showed no significant associations with RF, anti‐CCP, ANA, or anti‐ENA antibodies (Table 3).

**TABLE 3:** Chikungunya ELISA IgM antibodies in rheumatoid arthritis patients by status of autoantibodies.

Autoantibodies	Abcam‐IgM				Euroimmun‐IgM			
	Positive (*n* = 7)	Gray zone (*n* = 14)	Negative (*n* = 77)	*p*	Positive (*n* = 2)	Borderline (*n* = 0)	Negative (*n* = 96)	*p*
RF (+)	5	12	58	0.663	2	0	73	0.429
RF (−)	2	2	19		0	0	23	
Anti‐CCP (+)	6	12	63	0.916	2	0	79	0.513
Anti‐CCP (−)	1	2	14		0	0	17	
ANA ≧ 1:40 (+)	3	4	35	0.502	1	0	41	0.837
ANA ≧ 1:40 (−)	4	10	42		1	0	55	
ANA ≧ 1:80 (+)	1	1	26	0.088	1	0	69	0.498
ANA ≧ 1:80 (−)	6	13	51		1	0	27	
Anti‐ENA (+)	0	1	11	0.446	0	0	12	0.594
Anti‐ENA (−)	7	13	66		2	0	84	

Abbreviations: ANA, antinuclear antibody; anti‐CCP, anti‐cyclic citrullinated peptide; anti‐ENA, anti‐extractable nuclear antigen; RF, rheumatoid factor.

### 3.4 Agreement Between Neutralization‐IgG and ELISA IgG Results

Cohen's *κ* was 0 for both Abcam‐IgG versus neutralizing‐IgG and Euroimmun‐IgG versus neutralizing‐IgG, with an overall agreement of 98% for each comparison (Table 4). Although the overall agreements were high, the *κ* values were 0, indicating no agreement beyond chance. The positive agreements of 0% highlight a complete lack of concordance in identifying positive cases in these assays.

**TABLE 4:** Agreement between neutralizing‐IgG and ELISA‐IgG results in rheumatoid arthritis patients.

	Neutralizing‐IgG		Positive agreement % (95% CI)	Negative agreement % (95% CI)	Overall agreement % (95% CI)	*κ*
	Positive (*n* = 2)	Negative (*n* = 96)				
Abcam‐IgG						
Positive (*n* = 0)	0	0	0.0 (0.0–65.8)	100 (96.2–100.0)	98.0 (92.9–99.4)	0
Negative (*n* = 98)	2	96				
Euroimmun‐ IgG						
Positive (*n* = 0)	0	0	0.0 (0.0–65.8)	100 (96.2–100.0)	98.0 (92.9–99.4)	0
Negative (*n* = 98)	2	96				

Abbreviations: CI: confidence interval; κ: Cohen's kappa.

### 3.5 Agreement Between Abcam‐IgM and Euroimmun‐IgM ELISA Kits

For IgM assays, the positive agreement between the two kits was 0%, the negative agreement was 91.5%, and the overall agreement was 89.3%. The *κ* value was –0.38, indicating poor concordance (Table 5).

**TABLE 5:** Agreement between Abcam‐IgM and Euroimmun‐IgM results in rheumatoid arthritis patients.

	Euroimmun‐IgM		Positive agreement % (95% CI)	Negative agreement % (95% CI)	Overall agreement % (95% CI)	*κ* (95% CI)
	Positive	Negative				
Abcam‐IgM						
Positive (*n* = 7)	0	7	0.0 (0.0–65.8)	91.5 (83.4–95.8)	89.3 (80.9–94.3)	−0.38 (−0.081–0.004)
Negative (*n* = 77)	2	75				

Abbreviations: CI, confidence interval; *κ*, Cohen's kappa.

### 3.6 Clinical Profiles of Neutralizing Antibody‐ Positive RA Patients

Both RA patients with detectable neutralizing antibodies were RF‐positive and anti‐CCP‐positive. ANA titers were elevated (1:160 and 1:40), and anti‐ENA tests were negative. *HLA‐DR* genotyping revealed *DR9/DR12* in one patient and *DR15/DR15* in the other. Radiography showed bone erosion in one patient and joint‐space narrowing in both. Both patients were treated with hydroxychloroquine, sulfasalazine, and methotrexate; one also received abatacept (Table 6).

**TABLE 6:** Clinical characteristics of rheumatoid arthritis patients with positive Chikungunya neutralizing antibodies.

	Patient 1	Patient 2
Anti‐chikungunya Ab		
Abcam‐IgG	N	N
Abcam‐IgM	N	N
Euroimmun‐IgG	N	N
Euroimmun‐IgM	N	N
Neutralizing Ab	P	P
RF	P	P
Anti‐CCP	P	P
ANA titer	1:160	1:40
Anti‐ENA	N	N
*HLA‐DR*	*DR9/DR12*	*DR15/DR15*
X‐ray findings		
Bone erosion	Present	Absent
Joint space narrowing	Present	Present
Treatment		
DMARDs	Hydroxychloroquine, sulfasalazine, methotrexate	Hydroxychloroquine, sulfasalazine, methotrexate
Biologics	Abatacept	None

Abbreviations: Ab, antibody; ANA, antinuclear antibody; anti‐CCP, anti‐cyclic citrullinated peptide; anti‐ENA, anti‐extractable nuclear antigen; DMARDs, disease‐modifying antirheumatic drugs; N, negative; P, positive; RF, rheumatoid factor.

## 4 Discussion

In this study, we did not have robust evidence to show an association between CHIKV infection and the development of RA.

The presentation of chronic chikungunya arthritis in many patients could mimic RA clinically, and the pathogenesis of both diseases might be similar [23, 24, 25]. Besides sharing synovitis and bone erosion, elevated proinflammatory cytokines such as IL‐1β, IL‐6, IL‐17, and TNFα, etc. can be seen in the serum and synovium in both diseases [15, 26, 27]. Some differences also exist: RA patients have higher prevalences of RF and anti‐CCP antibody than those in chronic chikungunya arthritis patients [15].

Up to half of CHIKV‐infected patients might develop chronic chikungunya arthritis with some of these potentially developing RA later in life, fulfilling the 1987 ACR or 2010 ACR/EULAR classification criteria [22, 28, 29, 30]. In this study, two RA patients were found to be neutralizing antibody‐positive, indicating a previous CHIKV infection. One of these two RA patients had lived in Zhuhai City, Guangdong Province, for more than 10 years, a province in southern China with a history of local outbreaks of chikungunya, and had come back to Taiwan due to the onset of polyarthritis. The other patient had a travel history to Thailand, an endemic area. Both cases had positive RF, anti‐CCP, ANA, and typical radiographic findings. Similar findings can also be demonstrated in some patients with chronic chikungunya arthritis [30].

The linkages between chronic chikungunya arthritis and immune‐related genes have been demonstrated [31]. Bouquillard et al. [30] demonstrated that two‐thirds of RA patients following CHIKV infection have HLA‐DRB1*04 or 01. Our previous study also suggested that the HLA‐DR4 allele is related to the development of RA in Taiwan [32]. Thanapati et al. [33] revealed that the patients with DRB1*04/DQB1*03 haplotype are susceptible to CHIKV infection; however, neither of these two neutralizing antibody‐positive RA patients had HLA‐DR4. Owing to the small sample size of neutralizing antibody‐positive RA patients, the association between HLA genotype and the development of RA after CHIKV infection could not be established.

In this study, we detected chikungunya antibody using various methodologies and compared them. We used the viral neutralizing antibody as the reference standard [21], which has a specificity and sensitivity of 100%. We also performed tests using the Euroimmun and Abcam kits for both IgM and IgG. It is known that the Euroimmun‐IgM test for chikungunya tends to cross‐react with dengue virus, Zika virus, and other non‐chikungunya alphaviruses more readily than the IgG test [34, 35]; it is possible that similar results can be seen for the Abcam‐IgM and ‐IgG kits. This hypothesis is supported by our RT‐PCR results, which revealed that no CHIKV RNA could be detected in samples with positive IgM. Moreover, false‐positive and false‐negative results of the chikungunya ELISA IgM test might occur in patients with positive RF, as shown in the IBL CHIKV ELISA test manual. The sensitivity and specificity of the Euroimmun ELISA‐IgG test are also lower than those of the neutralizing test, as indicated in the instruction manual, while the Abcam ELISA‐IgG test may also yield similar results. The interpretation of the Chikungunya ELISA test should therefore be performed carefully. In this study, the positivity of Abcam‐IgM and Euroimmun‐IgM does not relate to the presence of RF. This study showed an extremely poor agreement (Cohen's *κ* = 0) between neutralizing antibody and Abcam‐IgG or Euroimmun‐IgG, although they had high overall agreements, which were caused by high negative agreements, a common finding in a very low‐prevalence area. A similar finding could also be found between Euroimmun‐IgM and Abcam‐IgM. The discordances between ELISA and neutralization assays appear related to cross‐reactions among alphaviruses, with arboviruses, and nonspecific reactions in autoimmune disease patients.

There are two major limitations in this study. First, the low prevalence of CHIKV‐neutralizing antibody positivity limits the statistical power to establish an association between CHIKV infection and the development of RA. Second, the cross‐sectional nature of this study masks the chronological information between exposure and outcome and prevents causal inference. Because Taiwan is a non‐endemic area for chikungunya infection, it is difficult to solve these two problems.

In summary, this study does not have enough power to conclude that CHIKV infection might be an environmental trigger factor in RA development in Taiwan, although a potential association between CHIVK infection and RA cannot be excluded. The results in this study are preliminary and are merely at the hypothesis‐generating stage in an attempt to investigate the possible relationship between CHIKV infection and RA. The discrepancies among serologic assays underscore the importance of confirmatory testing, such as a neutralizing antibody test, and careful interpretation is needed, especially in patients with autoimmune diseases.

## Author Contributions

Chang‐Yi Yen: writing – original draft, formal analysis, investigation. Chang‐Chi Lin: validation, methodology, data curation. Ping‐Chang Lin: methodology, formal analysis, data curation. Ching‐Yi Tsai: methodology, data curation. Li‐Teh Liu: methodology, formal analysis. Chen‐Hsuan Lin: validation, formal analysis. Chun‐Hong Chen: methodology, conceptualization. Chia‐Chun Tseng, Hua‐Chen Chan, Cheng‐Chin Wu, and Tsan‐Teng Ou: investigation. Jih‐Jin Tsai: writing – review and editing, visualization, validation, resources, funding acquisition, conceptualization. Jeng‐Hsien Yen: supervision, conceptualization.

## Conflicts of Interest

The authors declare no conflicts of interest.

## Data Availability

The data of this study are available from the corresponding author upon reasonable request.
